# Defining the Factors That Contribute to On-Target Specificity of Antisense Oligonucleotides

**DOI:** 10.1371/journal.pone.0101752

**Published:** 2014-07-29

**Authors:** Walt F. Lima, Timothy A. Vickers, Josh Nichols, Cheryl Li, Stanley T. Crooke

**Affiliations:** Isis Pharmaceuticals Inc., Carlsbad, California, United States of America; German Cancer Research Center, Germany

## Abstract

To better understand the factors that influence the activity and specificity of antisense oligonucleotides (ASOs), we designed a minigene encoding superoxide dismutase 1 (SOD-1) and cloned the minigene into vectors for T7 transcription of pre-mRNA and splicing in a nuclear extract or for stable integration in cells. We designed a series of ASOs that covered the entire mRNA and determined the binding affinities and activities of the ASOs in a cell-free system and in cells. The mRNA bound known RNA-binding proteins on predicted binding sites in the mRNA. The higher order structure of the mRNA had a significantly greater effect than the RNA-binding proteins on ASO binding affinities as the ASO activities in cells and in the cell-free systems were consistent. We identified several ASOs that exhibited off-target hybridization to the SOD-1 minigene mRNA in the cell-free system. Off-target hybridization occurred only at highly accessible unstructured sites in the mRNA and these interactions were inhibited by both the higher order structure of the mRNA and by RNA-binding proteins. The same off-target hybridization interactions were identified in cells that overexpress *E. coli* RNase H1. No off-target activity was observed for cells expressing only endogenous human RNase H1. Neither were these off-target heteroduplexes substrates for recombinant human RNase H1 under multiple-turnover kinetics suggesting that the endogenous enzyme functions under similar kinetic parameters in cells and in the cell-free system. These results provide a blueprint for design of more potent and more specific ASOs.

## Introduction

Antisense oligonucleotides (ASOs) have proven of value in determining gene functions and as a new therapeutic class [1]. Once ASOs bind via Watson-Crick hybridization to target RNAs, these agents may work through a variety of mechanisms of action [1]–[3]. DNA-like ASOs hybridized to target RNA create a substrate for cellular RNase H1 [4]. RNase H1 is ubiquitously expressed in prokaryotes and eukaryotes and is found in the nucleus, cytoplasm, and mitochondria of eukaryotic cells [5]–[10]. Consistent with the cellular distribution of RNase H1, DNA-like ASOs effectively target both exonic and intronic regions of pre-mRNAs as well as nuclear retained RNAs [11]–[12].

Numerous factors affect the potency and specificity of ASOs in cells including biostability, cellular uptake, subcellular distribution, protein interactions, and hybridization affinities for the target RNA [13]. The importance of hybridization is demonstrated by the correlation between hybridization affinity of the ASO and activity observed in cell assays and *in vivo* [14]–[18]. Hybridization stabilities are often determined by measuring the T_m_ of an ASO hybridized to length-matched complementary RNA [19]. Although measurements using simple duplex structures are useful for understanding nearest-neighbor effects and the influence of chemically modified nucleotides on ASO hybridization stability, these types of experiments cannot be used to quantify factors that affect the interaction between the ASO and target RNA in the cell such as the higher order structure of the RNA and RNA-binding proteins.

The secondary structure of the target RNA has been shown to significantly affect binding of ASOs [20]. For example, ASOs targeting the double-strand regions of a simple RNA hairpin structure have binding affinities three orders of magnitude weaker than ASOs targeting the single-strand region [20]. Computer algorithms have been developed to calculate secondary structures of target RNA and the free energies of ASO binding considering the effect of RNA secondary structure at the binding site [21]. These calculations correlate well with observed ASO activity for short target RNAs containing simple secondary structures. When compared to ASO activity in cells, however, the binding predictions are less correlative [22].

The roles of RNA-binding proteins on ASO activity are poorly understood. Several studies have shown that proteins can be recruited to the ASO using bifunctional RNA or peptide motifs [23]–[27]. ASOs containing RNA-like nucleotide modifications recruit double-strand RNA-binding proteins directly to the ASO/pre-mRNA heteroduplex [28]. However, these strategies do not address the impact of RNA-binding proteins present in cells. One study showed that ASOs are capable of competing with RNA-binding proteins for binding to the target RNA, although these observations were limited to a single class of proteins [28]. Specifically, high affinity, uniformly modified ASOs were used to effectively compete with hnRNPs for binding to pre-mRNA and redirected splicing of the pre-mRNA [28].

**Figure 1:**
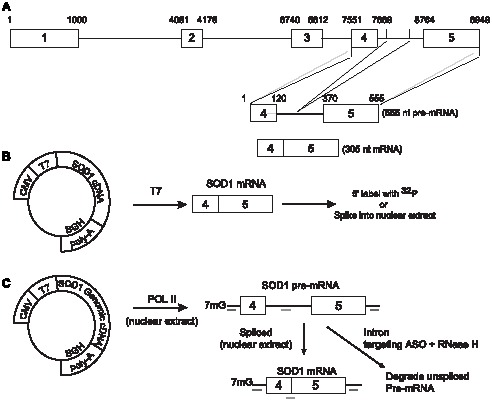
SOD-1 minigene construct. (A) The SOD-1 minigene contains the fourth and fifth exons of the SOD-1 gene and intervening intron. The intron was reduced to 250 nucleotides after the central 845 nucleotides were eliminated by PCR. The resulting minigene encodes a 555-nucleotide pre-mRNA and 305-nucleotide mRNA. (B) The minigene derived from the SOD-1 cDNA template was cloned into pcDNA3.1 vector containing both T7 and CMV promoters for, respectively, T7 and Pol II polymerase transcription and a BGH polyadenylation signal. The T7 transcribed SOD-1 minigene mRNA was PAGE purified and either 5′-labeled with ^32^P or spiked into the denatured nuclear extract. C) The SOD-1 genomic DNA derived SOD-1 minigene was cloned into the same vector and added to the nuclear extract for Pol II transcription and splicing into the mRNA. The unspliced pre-mRNA was degraded using an antisense ASO targeting the intron region and excess *E. coli* RNase H1. Levels of the SOD-1 minigene pre-mRNA and mRNA were quantified by qRT-PCR using primers complementary to the vector regions immediately upstream and downstream of the SOD-1 minigene RNA region (dark lines) and probes complementary to, respectively, the intron region or exon-exon junction (grey lines).

To better understand the molecular mechanisms of action of ASOs and the factors that influence potency and specificity, we created a superoxide dismutase 1 (SOD-1) minigene system that supports seamless evaluation of ASO effects on RNA in cell-free systems and in cells. The minigene system was designed to be large enough to contain an intron, coding regions, and 5′ and 3′-non-coding regions but small enough to be synthesized by T7 polymerase (Fig. 1). The minigene was cloned into vectors enabling Pol II transcription and splicing of the RNA in a nuclear extract or stable transfection into cells under transcriptional control by tetracycline. Thus this system provides a tool that supports the type of experimental control necessary to test mechanistic hypothesis. In this paper we report the evaluation and validation of the system, its use to characterize on- and off-target effects of ASOs with activity mediated by RNase H1, and impact of various factors on potency and specificity.

## Materials and Methods

### Preparation of SOD-1 minigene construct

A region of *SOD-1* comprised of the 117-nt-long exon 4, intron 4, and the 460-nt exon 5 was amplified from genomic DNA (Ref. Seq.: NG_008689.1). Deletion mutagenesis of 845 bp from the center of intron 4 was performed by overlap extension PCR according to Lee et al. [29] using primers specific for regions in exon 4 and exon 5 (E4-XhoI-FP: CAAACTCTCGAGAGGCATGTTGGAGACTTGG; E5-HindIII-RP: CTTTCAAAGCTTTTTAGTTTGAATTTGGATTCTT) and chimeric intron primers (I4-RP: ATTAGGAAATATCTCTCTACTAGGATTAAT; I4-FP: AGAGAGATATTTCCTAATTTGAACTGCAAG). The resulting PCR product containing 250 nt of intron 4 was then cloned into the vector pcDNA3.1 (-) at the with XhoI and HindIII sites to create pcDNA_SOD1.

### Preparation of antisense oligonucleotides

Synthesis and purification of phosphorothioate/2′-MOE oligonucleotides was performed using an Applied Biosystems 380B automated DNA synthesizer as described previously [30]. All ASOs were 20 bases in length with 2′-*O*-methoxyethyl substitutions at the positions 1–5 and 16–20 (gap-mers) or at every position (2′MOE). The ASOs contained either uniform phosphodiester linkages for use in the cell free assays or uniform phosphorothioate substitutions for evaluation in the cellular assay.

### Preparation of T7 transcribed SOD-1 minigene mRNA and oligoribonucleotide targets

T7 transcribed mRNA was generated from linearized DNA templates using a MEGAscript Kit according to the manufacturer's protocol (Life Technologies, Cat# AM1334M). Following a 5-h incubation at 37°C, 5 U of DNase I (Life Technologies) was added for 30 min at 37°C to remove any template DNA. After the DNase treatment, the reaction was adjusted to 300 mM sodium acetate, and extracted once with phenol/chloroform followed by a chloroform extraction. The RNA was then precipitated with 2 volumes of 100% ethanol.

The RNA was 5′-end labeled with ^32^P by first dephosphorylating the transcript using 50 µg of RNA and 10 µL of Antarctic Phosphatase (New England Biolabs, Cat# M0289) in 100 µL 1× Antarctic Phosphatase buffer. Reactions were incubated at 37°C for 60 min and heat inactivated at 65°C for 5 min. The dephosphorylated RNA was purified using an RNeasy Mini Kit as per the manufacturer's instructions (Qiagen, Cat# 74104). RNAs were 5′-end labeled using 40 pmol of RNA, 20 U of T4 polynucleotide kinase (Promega, Cat# M4101), 120 pmol [γ-^32^P] ATP (6000 ci/mmol) (Perkin Elmer, Cat# NEG035C005MC), 70 mM Tris, pH 7.6, 10 mM MgCl_2_, and 50 mM dithiothreitol. The kinase reaction was incubated at 37°C for 30 min and quenched using Gel Loading Buffer II (Life Technologies, Cat# AM8546G). The labeled RNAs were purified by gel electrophoresis on a 12% denaturing polyacrylamide gel as previously described [31].

### Binding affinities of ASOs for oligoribonucleotide targets and T7 transcribed SOD-1 minigene mRNA

ASO binding to the ^32^P-labeled T7 transcribed mRNA or to ^32^P-labeled oligoribonucleotide targets were determined by combining 1 nM mRNA with 100 nM ASO in hybridization buffer (20 mM Tris, pH 7.5, 50 nM NaCl, 2 mM MgCl_2_). Reactions were then heated to 90°C for 5 min and cooled to 37°C. To each reaction, 40 U of RNaseOUT (Life Technologies, P/N#100000840) and tris(2-carboxyethyl)phosphine) (TCEP; 0.2 mM final concentration) were added, and samples were incubated for 1 h. *E. coli* RNase H1 cleavage reactions were initiated by adding 25 U of enzyme (New England Biolabs) to a 100 µL reaction; samples were incubated at 37°C for 60 min. Reactions were quenched by adding Loading Buffer II, then heated to 90°C for 2 minutes and loaded onto a 6% denaturing polyacrylamide gel pre-heated to at least 50°C. Gels were analyzed using STORM 960 PhosphorImager (GE Lifesciences). The dissociation constants (K_d_) for ASOs bound to ^32^P labeled oligoribonucleotides and T7 transcribed SOD-1 minigene mRNA were determined as described above except that ASO concentrations of 0.1, 1, 10, 100, and 1000 nM were analyzed.

### On- and off-target binding affinities of ASOs for T7 transcribed SOD-1 minigene mRNA spiked into the denatured nuclear extract

Denatured HeLa nuclear extract (Promega) was prepared by heating extract at 95°C for 3 h with occasional vortexing, followed by sonication for 1 h. The ASO activity for the T7 transcribed mRNA spiked into the denatured nuclear extract was determined by adding 50 ng RNA to 20 µL denatured nuclear extract. After 1 h at 25°C, 5 U of *E. coli* RNase H1 and ASO to a final concentration of 10 nM were added. The reaction was allowed to proceed for 1 h at 30°C. The reaction was then subjected to DNA digestion for 30 min at 30°C. RNA was purified using an RNeasy 96-well plate (QIAGEN) according to manufacturers' instructions. RNA was analyzed by quantitative reverse transcription-PCR using StepOne Real-Time PCR System (Life Technologies). Primers used to quantify the mRNA were forward, 5′-AGACCCAAGCTGGCTAGCGT-3′, and reverse, 5′-GGCTGATCAGCGGTTTAAAC-3′; the probe spanned the exon 4/5 junction and the sequence was 5′-56-FAM/CTGGTGGTC/ZEN/CATGAAAAAGCAGATGACTTG/3IABkFQ-3′.

On-target ASO binding to the SOD-1 minigene mRNA spiked into the denatured extract was determined as described above with the exception that primers and probe designed to amplify and detect exon 5 were used: forward, 5′- TGC ATC ATT GGC CGC ACA CTG GTG GTC CAT -3′; reverse, 5′ – GGC TGA TCA GCG GTT TAA AC -3′; and probe, 5′-/56-FAM/CGC TGG AAG/ZEN/TCG TTT GGC TTG TGG/3IABkFQ/-3′. Off-target ASO binding was determined using primers and probe designed to amplify and detect exon 4: forward, 5′-AGA CCC AAG CTG GCT AGC GT -3′; reverse, 5′-TTC ATG GAC CAC CAGTGT GC -3′; and probe, 5'-/56-FAM/CA AAG ATG G/ZEN/T GTG GCC GAT G/3IABkFQ/-3′.

### On- and off-target binding affinities of ASOs for SOD-1 minigene mRNA transcribed and spliced in the nuclear extract

A minimum of 46 ng of run-off template was added to a 20 µL nuclear extract containing 16% HeLa Nuclear Extract (Promega), 36% (v/v) Buffer A (20 mM HEPES, pH 7.9, 100 mM KCl, 0.2 mM EDTA, 0.5 mM DTT, and 20% glycerol), and 3 mM MgCl_2._ The reaction was allowed to equilibrate at 30°C for 30 min prior to the addition of 0.4 mM rNTPs to initiate transcription. Reactions were incubated for an additional 2 h at 30°C to allow transcription and splicing, then 5 U of *E. coli* RNase H and ASO to a final concentration of 10 nM were added. RNase H cleavage was allowed to proceed for 1 h at 30°C. The reaction was then subject to DNA digestion using 5 U of DNase I (Life Technologies) for 30 min at 30°C. The RNA was purified and analyzed as described above. To determine the amount of pre-mRNA, the same forward and reverse primers were used as described above with a probe targeting the intron region: 5′-56- FAM/TAGTGATTA/ZEN/CTTGACAGCCCAAAGTTATCT/3IABkFQ-3′. ASO on- and off-target binding to the SOD-1 minigene mRNA transcribed and spliced in the nuclear extract was determined as described above for the mRNA spiked into the denatured nuclear extract.

### Human RNase H1 activity assay

Single and multiple turnover kinetics for human RNase H1 were determined by incubating either 2.7 or 400 ng, respectively, of human RNase H1 in reaction buffer (30% glycerol, 20 nM Tris, pH 7.5, 50 mM NaCl, 3 mM TCEP, 60 U RNaseOUT) for 1 h at 25°C. To 20 µL heat denatured nuclear extract, 50 ng of T7 SOD1 mRNA, 1000 nM ASO, and 1 µL of human RNase H1 in reaction buffer were added, and samples were incubated for 8 min at 37°C. Digestion reactions were analyzed by qRT-PCR as described above for the *E. coli* H1 experiments.

### Identification of nuclear extract proteins bound to the SOD-1 minigene mRNA

Capture and displacement of RNA binding proteins was performed using a 20-nucleotide 5′-biotin labeled RNA complementary to the T7-SOD1 mRNA containing an 18 nucleotide DNA linker (IDT). The capture RNA targeted the region immediately upstream of exon 4 of the SOD-1 minigene: 5′-/5Biosg//iSp18/ACGCUAGCCAGCUUGGGUCU-3′. Briefly, 1 nmol of the biotinylated RNA was mixed with 1 nmol of the T7-SOD1 mRNA in 1× siRNA buffer (Thermo Scientific) and heated at 95°C for 2 min, then slowly cooled to 37°C and incubated for 1 h. The duplex was then mixed with 100 µL of prewashed Dynabeads MyOne Streptavidin C1 (Invitrogen) with continuous rotation for 1 h to allow the duplex to bind to the beads. Subsequent washing steps were done according to the manufacturer's instructions. Duplexed RNA bound to beads was then incubated with a 75- µL mixture containing 1.6 mM MgCl_2_, 20 mM creatine phosphate, 500 µM ATP, 10 mM HEPES-KOH (pH 8), 17% (v/v) Buffer D (20 mM HEPES-KOH, pH 8, 20% (v/v) glycerol, 0.1 M KCl, 0.2 mM EDTA, 1 mM DTT), and 33% (v/v) HeLa nuclear extract. Mixtures were incubated with constant rotation for 40 min at 30°C. Beads were isolated by magnetic selection and washed three times with Buffer D containing 300 mM KCl, followed by a wash with Buffer D containing 100 mM KCl. The beads were resuspended in 50 µL Buffer D containing 100 mM KCl and 250 pmol ASO was added. The mixture was incubated at 30°C for 1 h to allow the ASO to displace the proteins from the hybridization site. The supernatants were collected, resolved on an SDS-polyacrylamide gel, and analyzed by western blot.

### Preparation of the TET-inducible system

To enable tetracycline-inducible expression in cells, the minigene was subcloned into the vector pcDNA 4/TO between HindIII and XbaI restriction sites. The region of interest from pcDNA_SOD1 was amplified with primer set SOD1-Hind3-F (taaactt AAGCTT TAA TAC GAC TCA CTA TAG GGA GAC) and SOD1-EcoRI-R (tcagattc GAATTC TTT AGT TTG AAT TTG GAT TCT TTT AAT AGC) to give pcSOD1/TO. The forward primer is complementary to the T7 promoter from pcDNA_SOD1 and incorporates a HindIII site, whereas the reverse primer was complementary to the 3′ end of exon 5 and included an EcoRI site.

T-REX-293 and T-REX-HeLa cells were purchased from Invitrogen and cultured in DMEM supplemented with 10% fetal calf serum, streptomycin (0.1 µg/mL), penicillin (100 U/mL), and blasticidin (5 µg/mL). Plasmid pcSOD1/TO was transfected into T-REX-293 cells using Effectene transfection reagent according to the manufacturer's protocol (Qiagen). Cell lines with stably integrated minigene were selected in DMEM media containing 250 µg/mL zeocin. Zeocin-resistant colonies were expanded and then tested for tetracycline-inducible expression by qRT/PCR.

pSOD/TO-DEL was created by deleting 34 nucleotides comprising the target sites for ASOs 440238, 440239, 440282, and 440283 from pSOD/TO using a QuikChange Lightning Site-Directed Mutagenesis Kit according to the manufacturer's protocol (Qiagen). The sense primer was 5'-AGGTCCATGAAAAAGCAGAAAGACAGGAAACGCTGG-3'; the antisense primer was 5'-CCAGCGTTTCCTGTCTTTCTGCTTTTTCATGGACCT-3'.

### ASO/siRNA treatment in cells and qRT/PCR

SOD/TO and SOD/TO-RHA cells were seeded in 96-well plates at ∼50% confluency then treated the following day with the indicated concentrations of ASO in Opti-MEM media (Invitrogen) containing 5 µg/mL Lipofectamine 2000 (Invitrogen) for 4 h as described previously [34]. Following transfection, cells were washed once with PBS, and growth media containing 1 µg/mL tetracycline was added to induce minigene transcription. After a 3 h incubation, total RNA was purified using an RNeasy 3000 BioRobot (Qiagen) and mRNA levels were assessed by qRT/PCR performed essentially as described elsewhere [35]. Briefly, 10 µL of total RNA was analyzed in a final volume of 50 µL containing 200 nM gene-specific PCR primers, 0.2 mM of each dNTP, 75 nM fluorescently labeled oligonucleotide probe, 5 µL RT-PCR buffer, 5 mM MgCl_2_, 2 U of Platinum Taq DNA Polymerase (Invitrogen), and 8 U of RNase inhibitor. Reverse transcription was performed for 30 min at 48°C followed by PCR (40 thermal cycles of 30 s at 94°C and 1 minute at 60°C) using an ABI Prism 7700 Sequence Detector (Applied Biosystems). To avoid artifacts based upon well to well variation in cell number, mRNA levels were normalized to the total amount of RNA present in each reaction as determined by Ribogreen assay (Invitrogen) [36]. Primers were designed to specifically amplify spliced or pre-mRNA with detection by the same probe. To avoid amplification of endogenous SOD1, primers included vector sequence unique to the minigene. The sequences of the primer/probe sets are listed in Table 1.

**Table 1:** Minigene specific qRT/PCR primers and probes.

primer	Sequence	Amplified fragment
E4FP	taa tac gac tca cta tag gga ga	Exon 4/vector
J45RP	CTG CTT TTT CAT GGA CCA CCA	Exon 4
E4PRB	CAA AGA TGG TGT GGC CGA TG	Exon 4
J45FP	TGG TGG TCC ATG AAA AAG CAG	Exon 5
E5RP	ctg tgc tgg ata tct gca gaa ttc TTT AG	Vector/Exon 5
E5PRB	CGC TGG AAG TCG TTT GGC TTG TGG	Exon 5

To avoid amplification of endogenous SOD-1, each primer/probe set includes vector sequence unique to the minigene (lower case). The exon 4 primer/probe set, E4 SPL, consists of E4FP, J45RP, and E4 PRB, whereas the exon 5 specific primer/probe set, E5 SPL, consists of J45FP, E5RP, and E5 PRB.

### RLM-RACE mapping of RNase H on- and off-target cleavage sites

SOD/TO and SOD/TO-RHA cells were seeded in 6-well plates at ∼50% confluency and treated the following day with 50 nM ASO in Opti-MEM media (Invitrogen) containing 5 µg/mL Lipofectamine 2000 (Invitrogen) for 4 h, as described above. Following transfection cells were washed once with PBS, then growth media containing 1 µg/mL tetracycline was added to induce minigene transcription. After a 3 h incubation, total RNA was purified using RNeasy mini columns (Qiagen). Total RNA was ligated to 1 µg FirstChoice 5′RACE Adapter RNA oligonucleotide (Applied Biosystems). The ligated products were reverse transcribed using random decamers and MMLV-RT according to the manufacturer's guidelines. The resulting cDNAs were amplified by successive rounds of PCR with nested pairs of primers specific either to exon 4 or to exon 5 of the SOD/TO minigene. The first round of PCR was performed for 30 cycles using the 5′RACE adaptor outer FP and either the Exon 5 outer RP (5′- GCAACTCTGAAAAAGTCACACAA-3′) or Exon 4 outer RP (5′- CACCAGTGTGCGGCCAATGAT-3′). The second round of PCR was performed for 30 cycles using the 5′RACE adaptor inner FP and either the Exon 5 inner RP (5′- TACAGCTAGCAGGATAACAGATGA-3′) or Exon 4 inner RP (5′-CAATGGTCTCCTGAGAGTGAGA-3′). RT-PCR production were separated on a 2% agarose gel in 1× TBE buffer and visualized by ethidium bromide staining. Ethidium bromide-stained PCR products were gel purified and cloned into pCR4-TOPO (Invitrogen) for sequence analysis to confirm putative ASO on- and off-target cleavage sites. For quantitative RACE (qRACE), cDNAs were amplified using an ABI Prism 7700 Sequence Detector as detailed above with the same outer primers with the addition of the probes for exon 4 (5′- FAM/CTGTGATCT/ZEN/CACTCTCAGGAGACCATTGCA/3IABkFQ-3′) or for exon 5 (5′- FAM/TTGGCTTGT/ZEN/GGTGTAATTGGGATCGCCCAA/3IABkFQ-3′).

## Results

### Preparation and characterization of the cell-free SOD-1 minigene system

To evaluate the factors that contribute to ASO activity, we prepared two minigene constructs from the *SOD-1* cDNA and from the *SOD-1* genomic DNA. Both contained sequences from exons 4 and 5 and the genomic construct also contained part of the intervening intron from which the central 845 nucleotides of were excised (Fig. 1A). The SOD-1 minigene constructs were then cloned into a vector containing both T7 and CMV RNA polymerase promoters and a bovine growth hormone (BGH) polyadenylation signal (Fig. 1B and C). The cDNA vector was used to prepare the “naked” SOD-1 minigene mRNA with T7 RNA polymerase. The naked mRNA was either 5′-end labeled with ^32^P or added into a denatured nuclear extract to determine ASO binding affinities to mature mRNA in the absence of proteins (Fig. 1B).

To evaluate the impact of nuclear protein binding to the mRNA on ASO binding affinity, the genomic SOD-1 DNA vector was incubated in a purified nuclear extract optimized for Pol II transcription and pre-mRNA processing (Fig. 1C). The transcription and splicing efficiencies of the SOD-1 minigene in the nuclear extract were determined by quantitative RT-PCR (qRT-PCR) using primers complementary to the vector sequences flanking the minigene to avoid amplification of endogenous *SOD-1* mRNA and probes complementary to either the intronic region for identification of the pre-mRNA or the junction region between exons 4 and 5 for identification of the mRNA (Fig. 1C). Cycle time (CT) values of 23 and 20 were observed, respectively, for the pre-mRNA and mRNA suggesting that approximately 10–15% of the pre-mRNA was processed into the mRNA (data not shown). After an incubation to allow transcription and splicing of the *SOD-1* mRNA, the nuclear extracts were treated with ASOs targeting the intronic region of the minigene and excess *E. coli* RNase H1 was added to degrade the pre-mRNA and signal due to pre-mRNA in the mRNA/protein binding assays (Fig. 1C). The intron targeting ASO/RNase H1 treatment effectively eliminated the pre-mRNA as no detectable amplification was observed by qRT-PCR using the pre-mRNA specific probe after this treatment (data not shown).

Proteins bound to the mRNA in the nuclear extract were identified using a pull down and displacement assay described in Figure S1A. This method not only enables the identification of the proteins bound to the SOD-1 minigene mRNA but also the protein binding site on the mRNA (Fig. S1B and C). The proteins bound included known RNA binding proteins that have been shown to be involved in mRNA processing and export of the mRNA from the nucleus (Fig. S1C) [37–45)]. The binding sites for these proteins were consistent with their reported binding specificities (Fig. S1C). For example, the hnRNP H and F proteins were bound to the SOD-1 minigene mRNA at the target sites for ASOs 19, 37, and 38, which contain the preferred guanosine-rich binding motifs for these proteins (Fig. S1B and C) [46]. In addition, proteins associated with the exon-junction complex (e.g., Magoh, UAF35, UAF56, Y14, and ALY) were identified at the reported binding site immediately upstream of the exon-exon junction (Fig. S1B and C) [40]–[48]. Finally, the splicing factors SF2 and SFRS5 bound at sites on the mRNA adjacent to or at the exon-exon junction (Fig. S1B and C) [40]–[41].

### Higher order structure of the SOD-1 minigene mRNA significantly influences ASO binding

**Figure 2:**
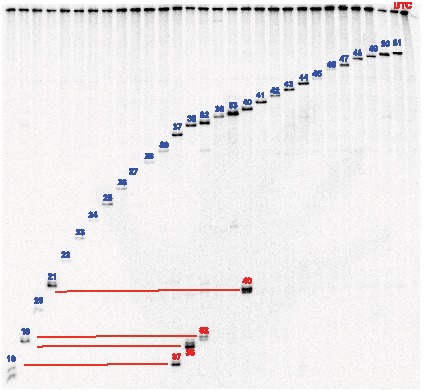
ASO binding to naked ^32^P-labeled SOD-1 minigene mRNA. Denaturing PAGE analysis of digestion reactions with ASOs and without ASO (labeled UTC). The bands corresponding to the RNase H1 cleavage products from on-target binding are labeled with ASO number in blue and off-target ASO cleavage products are labeled with red ASO numbers. The position of the off-target ASO hybridization in the SOD-1 minigene mRNA was determined by comparing the size of the off-target cleavage bands with the size of the on-target cleavage bands (joined by red lines).

The binding affinities for the 29 antisense oligonucleotides (ASOs) shown in Figure S1B were determined for the naked SOD-1 minigene mRNA as described in Figure S2A. Under these conditions, the amount of cleavage observed for each ASO is not limited by the enzymatic activity of *E. coli* RNase H1 but rather by the amount of heteroduplex formed. The observed cleavage products were consistent with the expected positions of ASO hybridization to the SOD-1 minigene mRNA (Fig. S1B and Fig. 2). The *E. coli* RNase H1 cleavage activity observed for each ASO/mRNA heteroduplex differed depending on the target site (Fig. 2). For example, greater *E. coli* RNase H1 cleavage activity was observed for the ASO 50, 51, and 83, heteroduplexes, whereas reduced cleavage activity was observed for ASOs 20 and 22 to 28 (Fig. 2). Given that the cleavage reactions were performed using the same concentration of ASO and excess *E. coli* RNase H1, the level of cleavage activity corresponds to the amount of ASO/mRNA heteroduplex formed. The differences in the amount of heteroduplex formed at each target site are likely not due to non-equilibrium conditions, as similar cleavage activities were observed for the various heteroduplexes incubated 1 to 48 hours prior to addition of the *E. coli* RNase H1 (data not shown).

**Figure 3:**
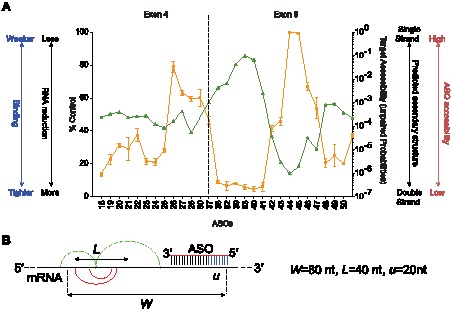
ASO binding to the SOD-1 minigene mRNA spiked into denatured nuclear extract. (A) The binding profile of the ASOs for the mRNA in denatured extract (orange line) compared with the predicted target site accessibilities (green line). ASO binding is reported as percent untreated mRNA control (left y-axis). A greater mRNA reduction (lower percent control) correlates with tighter ASO binding. Target site accessibilities are reported as unpaired probabilities (right y-axis). Greater probabilities predict that the target region is single stranded and accessible to ASO, and lower probabilities suggest that the target region is involved in mRNA structure. (B) Prediction of ASO target site accessibility within the SOD-1 minigene mRNA using RNAP-fold. The scanning window size (*w*) and maximum allowed distance between the base-pairs (*L*) within the mRNA were set to, respectively, 80 and 40 ribonucleotides. The binding accessibility of each ASO for the target mRNA was calculated based on the length of the ASO target site (*u* ), specifically 20 ribonucleotides.

ASO binding to the T7 transcribed SOD-1 minigene mRNA spiked into the denatured nuclear extract was determined using unlabeled SOD-1 minigene mRNA incubated in the denatured nuclear extract prior to the addition of the ASO and excess *E. coli* RNase H1 (Fig. S2B). Consistent with the naked 5′-^32^P labeled SOD-1 minigene mRNA, the *E. coli* RNase H1 cleavage activities for the mRNA added to the denatured nuclear extract varied significantly (Fig. 2 and 3A). Specifically, very little mRNA cleavage was observed for the 45 and 46 heteroduplexes. These experiments also showed that greater than 90% of the mRNA was full length and suggest that less than 10% of the mRNA was bound by these ASOs (Fig. 3A). Conversely, significantly greater cleavage activities were observed for ASOs 38 to 41 and 82 and 83. Incubation with these ASOs resulted in less than 10% full-length mRNA indicating that greater than 90% of the mRNA hybridized to ASO (Fig. 3A). One plausible explanation for the observed differences in the levels of heteroduplex formation is the higher order structure of the mRNA.

To evaluate this possibility, the folding algorithm RNAP-fold was used to predict base-pairing probabilities within the mRNA and consequently the accessibility of each ASO binding site in a scanning window approach (Fig. 3B). The predicted ASO target site accessibilities within the mRNA using this algorithm were consistent with the observed ASO binding activities (Fig. 3A). Specifically, the target sites for ASOs 38 to 41 and 82 and 83, which resulted in greater amounts of heteroduplex formation than other ASOs tested, are located in regions predicted to be single stranded (Fig. 3A). Conversely, a significantly lower percentage of the mRNA was hybridized with ASOs 45 and 46 whose target sites were predicted to be double-stranded and therefore less accessible to ASO binding (Fig. 3A). Taken together, the *in silico* predicted target site accessibilities and the binding profiles observed for the ASOs suggest that the differences in the amount mRNA bound to ASO is likely due to the higher order structure of the mRNA. In addition, the observed ASO binding profile appears to provide an accurate map of the higher order structure of the SOD-1 minigene mRNA.

The dissociation constants (K_d_s) for the ASOs binding to the SOD-1 minigene mRNA spiked in the denatured nuclear extract were determined by incubating the mRNA with increasing concentrations of ASO and treating the hybridization reactions with excess *E. coli* RNase H1 (Fig. S3). Greater than two-orders of magnitude differences in the dissociation constants were observed for the ASOs binding to different sites in the SOD-1 minigene mRNA (Fig. S3 and Table 2). For example, ASOs 18 and 40 exhibited the tightest binding affinities with K_d_s of 2 and 0.8 nM, respectively, whereas ASOs 26 and 44 exhibited the weakest binding affinities with K_d_s of 150 and 420 nM, respectively (Fig. S3 and Table 2). Thus, consistent with the *in silico* predicted target site accessibilities, the lowest affinity ASOs bound to sites predicted to be double stranded (Fig. 3A, Fig. S3 and Table 2).

**Table 2:** Binding affinities of ASOs for the oligoribonucleotide targets and SOD-1 minigene mRNA spiked into the nuclear extract.

ASO	SOD-1 mRNA K_d_ (nM)	Oligoribonucleotide K_d_ (nM)	ΔK_d_
18	2±0.3	0.36±0.05	6
19	4±1.4	0.27±0.07	15
20	5±0.5	0.31±0.01	16
27	40±3.3	0.19±0.03	210
37	10±2.1	0.26±0.02	38
38	2±0.8	0.28±0.06	7
40	0.8±0.3	0.37±0.07	2
45	420±6.2	0.22±0.04	1909
82	2±0.8	0.49±0.06	4

Dissociation constants (K_d_) were determined as described in the Materials and Methods and figure S3. Differences in the binding affinities (ΔK_d_) between the two targets were calculated by dividing the K_d_ of the ASOs for the SOD-1 minigene mRNA spiked into the denatured nuclear extract by the K_d_ for the oligoribonucleotide targets.

To better understand the effect of higher order structure in the mRNA on ASO binding, we determined the binding affinities of the ASOs for complementary oligoribonucleotides (Table 2). Similar binding affinities (K_d_s) were observed for all ASO/oligoribonucleotide heteroduplexes suggesting that the complementary oligoribonucleotides did not form secondary structures. The K_d_s for the ASO/oligoribonucleotide pairs varied by approximately 2 fold. In contrast, the range was greater than 100-fold for the same ASOs binding to the SOD-1 minigene mRNA (Table 2). Impressively, an almost 2000-fold difference was observed for ASO 44 binding to the SOD-1 minigene mRNA compared to the complementary oligoribonucleotide (Table 2). The ASOs with the tightest binding affinities for the mRNA (18, 40 and 44) had K_d_ values that were 2- to 6-fold weaker than those for the complementary oligoribonucleotides suggesting that these sites, although more accessible, have more competing structure than the oligoribonucleotide targets (Table 2). Taken together, these data suggest that the higher order structure of the SOD-1 minigene mRNA has a profound effect on the binding affinity of ASOs.

### RNA binding proteins have a modest effect on ASO binding to the SOD-1 minigene mRNA

**Figure 4:**
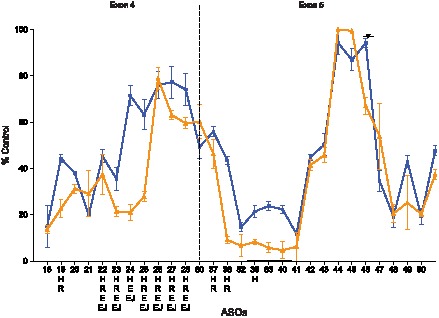
ASO binding to the SOD-1 minigene mRNA transcribed and spliced in the nuclear extract. The binding profile of the ASOs to mRNA transcribed and spliced in the nuclear extract (blue line) compared with the mRNA in denatured extract (orange line). The proteins bound to the mRNA at each ASO target site are listed in Figure S1. Here, proteins bound are listed below the ASO number by class: RNA-binding proteins (R), E-complex proteins (E), H-complex proteins (H) and exon-junction proteins (EJ). ASO binding is reported as percent untreated mRNA control. The mean and errors reported are based on three trials.

Next we evaluated ASO binding to the SOD-1 minigene mRNA that was transcribed and spliced in the nuclear extract (Fig. S2C). The binding profiles for the ASOs targeting the spliced mRNA were similar to those of the mRNA in the nuclear extract with the exception of those ASOs whose hybridization sites had were bound by proteins (Fig. S1C and Fig. 4). For example, less target RNA reduction was observed for the ASOs 19, 23, 24, 25, 27, 28, 38, and 39 when targeting the spliced SOD-1 minigene mRNA compared to the mRNA in denatured nuclear extract, suggesting that these ASOs were likely competing with the identified proteins for binding to the mRNA (Fig. S1C and Fig. 4). Specifically, the H-complex proteins, nucleolin and DHX36 bound to regions targeted by ASOs 19, 38, and 39 and the exon-junction and E-complex proteins bound to the ASO 23 to 28 binding sites (Fig. S1C and Fig. 4). ASOs 22, 26, and 37, which also target sites on the mRNA that were determined to contain bound proteins, reduced the spliced mRNA and the mRNA in the denatured nuclear extract to similar extents suggesting that the proteins bound to these sites had weaker binding affinities for the target RNA than did the ASOs (Fig. S1C and Fig. 4). Finally, ASOs 40, 46, and 83 reduced levels of the spliced mRNA less than the mRNA spiked into the denatured nuclear extract suggesting that either unidentified proteins were bound to these sites or the higher order structures were different under these two conditions (Fig. 4).

The K_d_s for the ASOs targeting transcribed and spliced SOD-1 mRNA are shown in Table 3. Consistent with the observed level of mRNA reduction for ASOs 19, 24, and 38 targeting protein binding sites in the spliced mRNA, 2 to 12-fold higher binding affinities were observed for these ASOs targeting the spliced mRNA compared to the mRNA spiked into the denatured nuclear extract (Fig. 4 and Table 3). These binding affinities demonstrate that the effect of RNA binding proteins on the binding affinity of ASOs for the target mRNA was significantly less than the influence of higher order mRNA structure on ASO binding (Tables 2 and 3). Taken together, these results suggest that although RNA binding proteins can compete with ASOs for target mRNA binding sites, the higher order structure of the mRNA has a greater deleterious effect on ASO binding than does protein binding. In addition, the observed similarities in the ASO binding profiles for the mRNA spiked into the denatured extract and the spliced mRNA indicate that these mRNAs are form similar higher order structures (Fig. 4).

**Table 3:** Binding affinities of ASOs for the SOD-1 minigene mRNA spiked into the denatured nuclear extract and mRNA transcribed and spliced in the nuclear extract.

ASO	Spliced SOD-1 mRNA K_d_ (nM)	Spiked SOD-1 mRNA K_d_ (nM)	ΔK_d_
18	3±2.4	2±0.3	1.5
19	8±1.1	4±1.4	2.0
20	6±0.3	5±0.5	1.2
24	47±5.9	4±1.6	11.8
37	16±2.2	10±2.1	1.6
38	8±1.5	2±0.8	4.0
40	3±0.6	0.8±0.3	3.8
82	4±0.8	2±0.8	2.0

Differences in the binding affinities (ΔK_d_) between the two targets were calculated by dividing the K_d_ of the ASOs for the SOD-1 minigene mRNA transcribed and spliced in the nuclear extract by the K_d_ for the SOD-1 minigene mRNA spiked into the denatured nuclear extract.

### Identification and characterization of ASO off-target site hybridization to the SOD-1 minigene mRNA

Analysis of the cleavage patterns for the 5′-^32^P labeled naked SOD-1 minigene mRNA showed that several ASOs induced more than one cleavage product; this suggests that certain ASOs hybridize to more than one site on the SOD-1 minigene mRNA (Fig. 2). For example, *E. coli* RNase H1 cleavage of ASOs 37, 38, 40, and 82 produced cleavage products consistent with ASO hybridization to the intended on-target site and additional lower molecular weight cleavage products (Fig. 2). Given that the SOD-1 minigene mRNA is 5′-labled, the lower molecular weights of the additional cleavage products indicate that the off-target hybridization sites are positioned upstream of the on-target sites.

The positions of the off-target binding sites were identified by matching the size of the off-target cleavage products with on-target cleavage products of approximately the same molecular weight. For example, the off-target cleavage product generated by ASO 40 migrated in the gel a similar distance as the on-target cleavage product generated by the ASO 21, suggesting that the off-target binding site for ASO 40 was in the vicinity of the on-target binding site for ASO 21 (Fig. 2). Similarly, the off-target binding sites for the ASOs 37, 38, and 82 were in the proximities of the on-target binding sites for ASOs 18 and 19 (Fig. 2). These predicted off-target sites involved ribonucleotide sequences that were at least partially complementary to the ASO sequences (Fig. S4A).

The algorithm RNAstructure 5.3 was used to predict the extent of base pairing in heteroduplex structures formed by ASOs 37, 38, 40 and 82 ASOs and their off-target binding site RNA sequences (Fig. S4B). These predicted heteroduplexes contained between 13 to 15 base pairs, and the predicted free energies for hybridization (ΔG°) were consistent with the number of base pairs formed, with the exception of the ASO 37 heteroduplex, which was significantly less stabile presumably as a result of a five nucleotide bulge (Fig. S4B). To determine whether the ASOs were binding to the off-target sites as predicted by the hybridization algorithm, RNA oligonucleotides corresponding to the off-target site sequences were prepared, 5′-labeled with ^32^P, and hybridized with the ASOs. The secondary structures of the heteroduplexes were mapped enzymatically using the single-strand specific endoribonucleases T1 and A (Fig. S4C). The RNase T1 and A cleavage patterns for the heteroduplexes were consistent with the structures predicted *in silico* (Fig. S4B and C). The enzymatic structure mapping of ASO 37, 38, 40, and 82 heteroduplexes suggested that these ASOs were capable of hybridizing to the predicted off-target binding sites. The binding affinities of ASOs 37, 38, 40, and 82 for the on- and off-target oligoribonucleotides are shown in Table 4. Consistent with the calculated free energies for hybridization, all of the ASOs tested exhibited 2 to 14-fold weaker binding affinities for the off-target compared to the on-target oligoribonucleotides (Table 4).

**Table 4:** Binding affinities of the ASOs for the on- and off-target oligoribonucleotides.

ASO	Off-target K_d_ (nM)	On-target K_d_ (nM)	ΔK_d_
37	0.6±0.08	0.26±0.02	2.3
38	3.7±1.3	0.28±0.06	13.2
40	2.9±0.9	0.37±0.07	7.8
82	1.1±0.2	0.49±0.06	2.2

Dissociation constants (K_d_) were determined as described in in the Materials and Methods and figure S3. Differences in the binding affinities (ΔK_d_) between the two targets were calculated by dividing the K_d_ of the ASOs for the off-target oligoribonucleotide by the K_d_ for the on-target oligoribonucleotide.

### Higher order structure of the mRNA and RNA binding proteins inhibit off-target ASO binding

**Figure 5:**
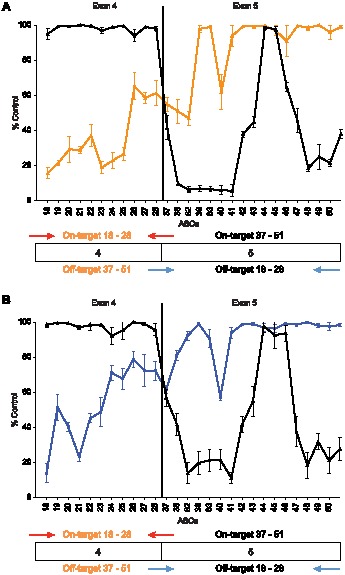
On- and off-target ASO binding to the SOD-1 minigene mRNA. (A) Orange line represents the ASO binding profile for the mRNA spiked into the denatured nuclear extract obtained using exon 4-specific primers (red arrows). Black line represents the ASO binding profile for the mRNA spiked into the denatured nuclear extract obtained using exon 5-specific primers (blue arrows). ASO binding is reported as percent untreated mRNA control. The mean and standard errors reported are based on three experiments. On-target reduction of mRNA was observed for ASOs 18–28 targeting exon 4 when the exon 4-specific primer/probe set was used (orange line). No reduction of the mRNA was observed for these ASOs using the exon 5 primer/probe set (black line) indicating that these ASOs did not exhibit off-target binding to the exon 5 region of the minigene mRNA. On-target reduction of mRNA was observed for ASOs 37–51 targeting exon 5 when the exon 5-specific primer/probe set was used (black line). Reduction of mRNA was observed for ASOs 37, 38, 40, and 82 using the exon 4-specific primer/probe set (orange line) indicating that these ASOs exhibited off-target binding to the exon 4 region of the minigene mRNA. (B) Blue line represents the ASO binding profile for the mRNA transcribed and spliced in the nuclear extract using exon 4-specific primers (red arrows). Black line represents the ASO binding profile for the mRNA transcribed and spliced in the nuclear extract using exon 5-specific primers (blue arrows). ASO binding is reported as percent untreated mRNA control. The mean and standard errors are based on three experiments. On-target reduction of mRNA was observed for ASOs 18–28 targeting exon 4 when the exon 4-specific primer/probe set was used (blue line). No reduction of the mRNA was observed for these ASOs using the exon 5 primer/probe set (black line) indicating that these ASOs did not exhibit off-target binding to the exon 5 region of the minigene mRNA. On-target reduction of mRNA was observed for ASOs 37–51 targeting exon 5 when the exon 5-specific primer/probe set was used (black line). Reduction of mRNA was observed in the presence of ASOs 37, 38, 40, and 82 using the exon 4-specific primer/probe set (blue line) indicating that these ASOs exhibited off-target binding to the exon 4 region of the minigene mRNA.

The off-target site binding of the ASOs to the SOD-1 minigene mRNA spiked into the denatured nuclear extract were determined using primer-probe sets that amplify the specific regions containing the on- and off-target sites. For example, the primer-probe set designed to amplify exon 4 was used to measure the on-target cleavage activities for ASOs 18–28 and off-target activities for ASOs 37–51 and the primer-probe set designed to amplifying exon 5 was used to measure the on-target cleavage activities for ASOs 37–51 and off-target activities for ASOs 18–28 (Fig. 5A). Consistent with the off-target binding observed for the ^32^P-labled mRNA, only ASOs 37, 38, 40, and 82 exhibited off-target binding to the SOD-1 minigene mRNA spiked into the denatured nuclear extract (Fig. 5A). ASOs 37, 38, 40, and 82 resulted in approximately 40–50% reduction of the mRNA when cleavage at the off-target sites were evaluated (Fig. 5A).

The dissociation constants (K_d_) for the off-target binding of ASOs 37, 38, 40, and 82 to the SOD-1 minigene mRNA spiked into the denatured nuclear extract were determined using the primer-probe set amplifying exon 4 (Fig. S3 and Fig. 5A). Approximately 2 to 50-fold weaker binding affinities were observed for the off-target site binding than for the on-target sites (Table 5). Interestingly, the approximately 2-fold difference between the binding affinities (ΔK_d_) for the on- and off-target hybridization of ASO 37 to the SOD-1 minigene mRNA spiked into the denatured nuclear extract was significantly less than the 14-fold ΔK_d_ observed for ASO 37 binding to the on- and off-target oligoribonucleotides (Tables 4 and 5). Differences in accessibility of the on- and off-target sites in the full-length mRNA may explain the smaller ΔK_d_. The off-target binding site for ASO 37 is positioned at approximately the on-target binding site for ASO 18, which exhibited a K_d_ of 2 nM for the SOD-1 minigene mRNA spiked into the denatured nuclear; the on-target K_d_ for ASO 37 is 10 nM (Table 2). The 5-fold tighter K_d_ observed for the on-target binding of ASO 18 compared to that for ASO 37 suggests that the hybridization site for ASO 18 is more accessible than the ASO 37 binding site and, consequently, is also more accessible for off-target binding (Table 5).

**Table 5:** Binding affinities of the ASOs for the on- and off-target sites of the SOD-1 minigene mRNA spiked into the denatured nuclear extract.

ASO	off-target K_d_ (nM)	On-target K_d_ (nM)	ΔK_d_
37	18±2.8	10±2.1	1.8
38	17±3.4	2±0.8	8.5
40	37±6.5	0.8±0.3	46.3
82	10±1.7	2±0.8	5.0

Dissociation constants (K_d_) were determined as described in in the Materials and Methods and figure S3. Differences in the binding affinities (ΔK_d_) between the two targets were calculated by dividing the K_d_ of the ASOs for the off-target sites by the K_d_ for the on-target sites.

Consistent with the off-target binding activities observed for the ^32^P-labeled mRNA and the mRNA spiked into the nuclear extract, only ASOs 37, 38, 40, and 82 exhibited off-target binding to the SOD-1 minigene mRNA spliced in the nuclear extract (Fig. 5B). Similar RNA reductions were observed for the off-target binding of ASOs 37 and 40 to the mRNA either spliced in the nuclear extract or spiked into the denatured nuclear extract (Fig. 5A and B). Conversely, the off-target binding observed for ASOs 38 and 82 ASOs were less pronounced for the spliced SOD-1 minigene mRNA than for the mRNA spiked into the denatured nuclear extract (Fig. 5B).

Weaker off-target binding affinities were observed for ASOs 37, 38, 40, and 82 to the mRNA spliced in the nuclear extract compared to the mRNA spiked into the denatured nuclear extract (Tables 5 and 6). Importantly, significantly weaker off-target binding was observed for ASOs 38 and 82, which target the ASO 19 site also shown to bind proteins (Fig. S1C and Table 6). Interestingly, the RNA binding proteins appeared to exhibit a significantly greater effect on the off-target binding of ASOs 38 and 82 than on the on-target binding (Tables 3 and 6). These data suggest that the off-target affinities observed for the ASOs 38 and 82 are inadequate to effectively compete with proteins for binding to the mRNA, whereas the binding affinity of the fully complementary ASO 19 ASO was sufficient to effectively compete with RNA binding proteins.

**Table 6:** Binding affinities of the ASOs for the on- and off-target sites of the SOD-1 minigene mRNA transcribed and spliced in the nuclear extract.

ASO	Off-target K_d_ (nM)	On-target K_d_ (nM)	ΔK_d_
37	51±6.3	16±2.2	3
38	221±16.7	8±1.5	28
40	44±2.7	3±0.6	15
82	672±22.3	4±0.8	168

Dissociation constants (K_d_) were determined as described in in the Materials and Methods and figure S3. Differences in the binding affinities (ΔK_d_) between the two targets were calculated by dividing the K_d_ of the ASOs for the off-target sites by the K_d_ for the on-target sites.

### Human RNase H1 enhances the target specificity of ASOs

Given that human RNase H1 has been shown to be responsible for ASO-mediated cleavage of target RNA in human cells, we determined the human RNase H1 cleavage activity for the on- and off-target ASO/mRNA heteroduplexes using recombinant enzyme (Fig. S2D) [4]. To ensure that the cleavage activities were exclusively due to the recombinant RNase H1, the measurements were performed using the denatured nuclear extract as the native extract was shown to contain human RNase H2 (data not shown). In addition, the cleavage activities were determined with the human RNase H1 concentration in excess of the heteroduplex substrate concentration (single-turnover conditions), similar to the conditions used for the *E. coli* enzyme, or with the heteroduplex substrate concentration in excess of the human RNase H1 concentration (multiple-turnover conditions) (Fig. S2D). Finally, to ensure that the mRNA was completely hybridized with the ASOs prior to addition of the enzyme, 1 µM ASO concentration was used (Fig. S2D).

As evaluated by on- or off-target-specific qRT-PCR, the SOD-1 minigene mRNA hybridized with ASOs 37, 38, 40, or 82 was fully degraded (e.g., 100% reduction) when using excess *E. coli* RNase H1 (Table S1). These results indicate that 100% of the mRNA was hybridized with the ASOs at their respective on- and off-target sites (Table S1). Slightly less SOD-1 minigene mRNA was degraded in the presence of excess human RNase H1 than in the presence of excess *E. coli* enzyme, particularly for the off-target site heteroduplexes (Table S1). Given that 100% of the mRNA was shown to be bound with ASO, the reduction in cleavage activity observed for the off-target heteroduplexes suggests that the human enzyme less effectively cleaves the off-target heteroduplexes than does *E. coli* RNase H1; presumably the mismatched base pairs have a greater inhibitory effect on the human enzyme.

Under multiple turnover conditions (i.e., heteroduplex substrate in excess of the human RNase H1), approximately 75 to 90% reduction of the SOD-1 minigene mRNA spiked into the denatured nuclear extract was observed in the presence of ASOs 37, 38, 40, and 82 at their respective on-target sites (Table S1). Human RNase H1 appeared to be unable to cleave the off-target heteroduplexes, as no reduction of the mRNA was observed due to hybridization of ASOs 37, 38, 40, and 82 to their respective off-target sites (Table S1).

### On-target but not off-target ASO activity was observed for SOD1 minigene in human cells

To evaluate ASO activity in human cells, the SOD1 minigene was cloned into the TET-regulated vector pcDNA 4/TO [49]. T-Rex 293 cells stably expressing the construct were evaluated for TET-inducible expression of the minigene by qRT/PCR using the primers/probes specific for spliced or pre-mRNA. Up-regulation in levels of pre-mRNA was detected almost immediately following addition of TET with maximal expression of approximately 300% of the uninduced control 45–60 minutes after addition of TET to the media (data not shown). Detectable levels of spliced mRNA lagged synthesis of the pre-mRNA by approximately 15 minutes; however, induction was at least 20-fold and did not plateau until almost 4 hours after addition of TET (data not shown).

**Figure 6:**
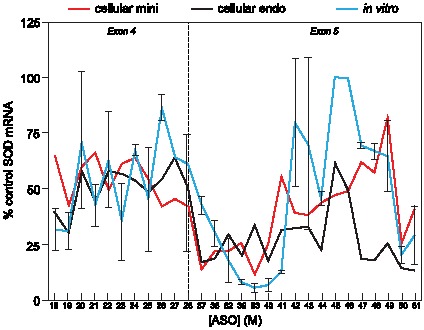
A) ASO activity for tetracycline-inducible SOD-1 minigene. Exons 4 and 5 and a truncated intron 4 were cloned into the vector pcDNA4/TO allowing for tetracycline regulated expression of the minigene and zeocin selection of stable cell lines. SOD/TO cells were transfected with ASOs. Following ASO treatment reduction of SOD1 minigene or endogenous SOD1 was evaluated by qRT/PCR using primers and probes specific for the minigene or endogenous SOD1. Data are presented as percent expression spliced mRNA relative to mock-treated control cells. The error bars represent the mean and standard errors of at least three experiments.

Next we determined activities of ASOs targeting the SOD-1 minigene RNA (Fig. S1B). To avoid possible false positives due to the presence of the ASO during PCR amplification, the exon 5 primer/probe set (E5 SPL) was used for ASOs directed to the exon 4 sequence, and the exon 4 primer/probe set (E4 SPL) was used for ASOs complementary to exon 5. ASO-mediated cleavage of the endogenous SOD1 message was evaluated using a primer/probe set specific to exon 3 of SOD1. Patterns of mRNA reduction for the endogenous SOD1 were similar to that of the minigene, with slightly more activity observed in exon 4 and in exon 5 with ASOs 47–52 (Fig. 6). The activity profile for the ASOs targeting the SOD-1 minigene in cells was similar to the binding profile observed for the SOD-1 minigene mRNA that was transcribed and spliced in the nuclear extract suggesting that the ASOs exhibited similar binding affinities for both mRNAs (Fig. 6). Given that the higher order structure of the mRNA appears to be the predominate factor influencing ASO binding to the mRNA, the similarities between the ASO activity in cells and ASO binding in the nuclear extract suggest that the mRNAs exhibit similar higher order structures (Fig. 6).

**Figure 7:**
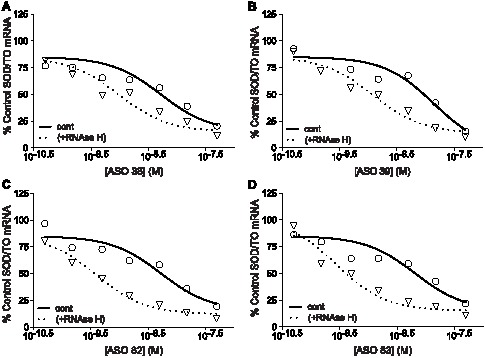
Effect of *E. coli* RNase H overexpression on on-target antisense activity. HEK 293 cells harboring the SOD/TO minigene or the SOD/TO minigene and pcDNA3.1-RHA were treated with ASOs at concentration between 0.5 and 150 nM. Following transfection and TET induction of the minigene, target RNA reduction was measured by qRT/PCR. Data are presented as percent mock-transfected control for SOD/TO (solid line) and SOD/TO-RHA cells (dashed line).

**Figure 8:**
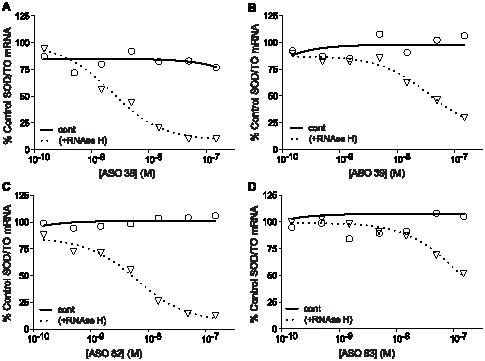
Effect of *E. coli* RNase H overexpression on off-target antisense activity. HEK 293 cells harboring the minigene or the SOD 282_DL minigene and pcDNA3.1-RHA were treated with ASOs at concentrations between 0.5 and 150 nM. Following transfection and TET induction of the minigene, target RNA reduction was determined by qRT/PCR. Data are presented as percent mock-transfected control for SOD 282_DL (solid line) and SOD 282_DLH cells (dashed line).

To explore off-target activity in cells, the on-target sites for ASOs 38, 39, 82, and 83 were deleted from the SOD/TO minigene by site-directed mutagenesis. A stable 293 cell line harboring the mutated minigene construct, SOD 282_DL, and a second cell line that overexpresses *E. coli* RNase H (SOD 282_DLH) were treated with ASOs at concentrations between 0.5 and 150 nM. As a control, SOD/TO cells with and without RNase H overexpression were treated with the same ASOs (Fig. S5). All ASOs displayed similar levels of activity in the SOD/TO cell line with IC_50_s ranging from 5 to 15 nM (Fig. 7, solid lines). In the presence of *E. coli* RNase H, the potencies of the ASOs increased by 5–10 fold (Fig. 7, dashed lines). No activity was observed for any ASOs targeting the deleted region in the SOD 282_DL cells, suggesting that these sequences promote little or no off-target activity (Fig. 8, solid lines). However, in agreement with our nuclear extract results, off-target activity was observed for each of these ASOs when *E. coli* RNase H was overexpressed (Fig. 8, dashed lines). ASO 38 showed the greatest off-target activity under these conditions with an IC_50_ just 2-fold greater than that observed for the on-target activity in the presence of excess RNase H. The off-target activity of ASO 82 was approximately 3-fold less than that of 38 while the activity of ASOs 39 and 83 was 16 and 44-fold less respectively. Similar results were obtained when human RNase H1 was overexpressed in the same cell lines (Fig. S6), suggesting that the limiting levels of endogenous RNase H1 are a major contributor to ASO specificity in mammalian cells.

**Figure 9:**
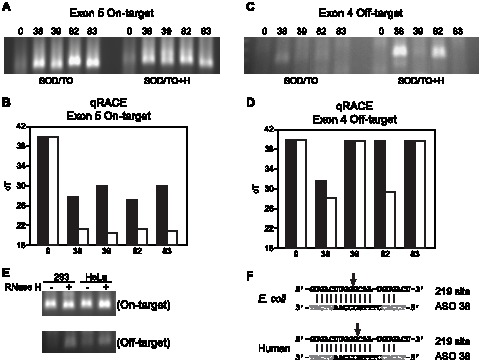
Cellular off-target cleavage is observed only with RNase H1 overexpression. A modified RLM-RACE protocol was used to determine sites of target-specific and off-target cleavage. A) Exon 5 target-specific RACE cleavage products. (B) Quantitative RACE of exon 5 target-specific cleavage products for SOD/TO cells (black bars) and SOD/TO-RHA cells (gray bars). Results are given as threshold cycle (cT) for the amplification reaction with or without overexpression of RNase H. C) Exon 4 off-target RACE cleavage products. D) Quantitative RACE of exon 4 off-target cleavage products (cT). E) Human RNase H1 was overexpressed in HeLa SOD/TO cells by infecting with adenoviral human RNase H1 for 48 hours [4]. Cells were then treated with 50 nM ASO 38, and RLM_RACE was performed as described above. 293 cells with or without *E. coli* RNase H; HeLa cells with or without human RNase H1. F) RACE products were gel purified and sequenced using gene specific RACE primers. Arrows indicate the predominant cleavage site.

The sites of on- and off-target cleavage were confirmed by 5′RACE in SOD/TO cells with and without RNase H overexpression. RACE primers designed to the on-target region of exon 5 specifically amplified DNA fragments corresponding to the expected cleavage site for each ASO (Fig. 9A) as confirmed by sequencing of the RACE PCR fragment. Quantitative RACE from the same experiment clearly demonstrated an increase in the amount of the cleavage products with overexpression of RNase H (Fig. 9B). Cleavage products were not detected for any ASO in control cells using RACE primers designed to the off-target region of exon 4 (Fig. 9C). However, as observed in the SOD 282_DL cell line, off target cleavage was detected in the presence of *E. coli* RNase H for ASO 38 and, to a lesser extent, ASO 82 (Fig. 9D). No off target cleavage products were detected for the other ASOs.

SOD/TO 293 cell lines overexpressing *E. coli* or SOD/TO HeLa cells infected with a human RNase H1 adenovirus vector were treated with ASO 38. Once again, no off-target cleavage products were detected in the absence of overexpression of RNase H; however, RACE cleavage products were clearly visible upon overexpression of either human or *E coli* RNase H (Fig. 9E). Interestingly, sequencing of RACE cleavage products revealed that the preferred off-target cleavage sites of these enzymes differed by one nucleotide (Fig. 9F). Taken together, these data suggest that although these ASOs are capable of binding to off-target sites on the SOD-1 minigene mRNA in cells, as detected in the presence of overexpressed RNase H, these binding interactions do not result in degradation of the mRNA in cells.

## Discussion

In this study we assessed the factors that influence the activity and specificity of ASOs. We developed a SOD-1 minigene system to better define the molecular mechanisms of action of ASOs in a cell-free environment and then extended our findings to the cell. The higher order structure of the SOD-1 minigene mRNA appeared to be the predominant factor affecting on-target ASO binding to the mRNA (Fig. 3A). ASOs targeting predicted double-stranded regions of the mRNA exhibited binding affinities at least three orders of magnitude lower than those targeting predicted single-stranded mRNA regions (Fig. 3A and Table 2). Interestingly, the ASOs with the tightest binding affinities for the SOD-1 minigene mRNA had affinities 2 to 6-fold lower than the binding affinities for unstructured oligoribonucleotide targets; this suggests that even the most accessible sites in the mRNA likely form higher order structures that inhibit ASO binding (Table 2). Given that both ASO binding to the mRNA and the secondary structure interactions within the RNA abide by the same principles of Watson-Crick base pairing, these findings should apply to all antisense oligonucleotides and to siRNAs. The magnitude of the difference in binding affinity between antisense oligonucleotides targeting single-stranded verses double-stranded regions of the mRNA will depend on the hybridization stabilities of the antisense oligonucleotides, which in turn is affected by length and chemical modifications of the ASO. The ASOs used here contained ten deoxyribonucleotide and ten 2′-methoxyethylribonucleotides, which when hybridized to RNA exhibit, respectively, lower or comparable hybridization stabilities to RNA-RNA base-pairs. The K_d_s measured for ASOs bound to the unstructured oligoribonucleotides ranged from 190 to 490 pM (Table 2). Consistent with these results, a previous study of antisense deca-oligoribonucleotides found three orders of magnitude differences in binding affinities for the ASOs targeting the single-stranded verses double-stranded regions of a hairpin target RNA [20]. Of course it is possible to mitigate the influence of higher order structure on ASO binding by using ASOs with higher affinity nucleotide modifications (e.g., 2′-constrained ethylribonucleotides), which more effectively compete with target RNA structure [50]. Alternatively, increasing the length of the ASOs would also enhance their hybridization stability (T_m_), although longer ASOs have higher likelihoods of encountering RNA structure and also have the potential to form self-structure [51]. A number of studies have found that the optimal length for an ASO is 17 to 20 nucleotides; the SOD-1 minigene system provides a clear rationale for this length restriction.

RNA binding proteins belonging to the H, E, and exon-junction complexes were shown to bind the SOD-1 minigene mRNA and appeared to modestly inhibit on-target ASO binding to the SOD-1 minigene mRNA (Fig. S1C, Fig. 4 and Table 3). In a previous study ASOs containing high affinity nucleotide modifications at all positions of the ASO were shown to effectively displace H-complex proteins from the pre-mRNA target to redirect splicing of the pre-mRNA [28]. Our results suggest that ASOs containing fewer high affinity nucleotide modifications also effectively compete with proteins for binding to the RNA. This indicates that binding affinities of the proteins for the mRNA are weaker than the affinities of ASOs. In fact, the reported binding affinities of hnRNPs for RNA were approximately two orders of magnitude weaker than the ASO binding affinities observed in this study (Table 3) [52]. The H-complex proteins bind mRNA as individual proteins, whereas the E and exon-junction complexes interact with larger regions of the mRNA via multiple proteins and therefore are expected to form more stable interactions with the RNA [37]–[41], [44]. Surprisingly, the proteins belonging to these complexes also exhibited only a modest effect on ASO binding (Fig. S1C, Fig. 4 and Table 3). It is not clear whether the proteins belonging to those complexes bind the SOD-1 minigene mRNA individually or as a complex, although the E-complex proteins do appear to form a competent complex, as splicing of the SOD-1 minigene mRNA was observed in the nuclear extract. Consequently, the observed influence of the E-complex proteins on ASO binding to the mRNA in the nuclear extract is expected to be reflective of conditions in the cell.

Off-target ASO hybridization to the SOD-1 minigene mRNA was observed for five of the 29 ASOs tested (Fig. 2 and 5). Importantly, all the off-target interactions involved regions within the SOD-1 minigene mRNA that were accessible to on-target ASOs and that were predicted to be single stranded (Fig. 3A and Fig. S4A). Specifically, the ASOs 18, 19, and 20 exhibited among the highest binding affinities for the mRNA with K_d_s ranging from 2 to 5 nM (Table 2) and the regions targeted by these ASOs were also bound by off-target ASOs. The off-target ASO/mRNA heteroduplexes formed between 13 and 15 base-pairs with the mRNA (Fig S4C). Surprisingly, off-target hybridization with less than 13 base pairs was not observed, even though statistically the number of potential off-target ASO sites increases as the number of complementary base pairs between the ASO and mRNA decreases. The stabilities of the off-target heteroduplexes, as determined using the unstructured oligoribonucleotide targets, were a modest 2 to 13-fold weaker compared to the on-target heteroduplexes with 20 base-pairs (Table 4). The lack of off-target ASO binding for heteroduplexes with less than 13 base pairs suggests that off-target hybridization requires binding affinities similar to on-target binding. In other words, modest reductions in binding affinity are sufficient to preclude off-target ASO hybridization to the RNA. These data also suggest that enhancing the binding affinity of the ASO for the target RNA using higher affinity modified nucleotides would likely also enhance the affinity of the ASO for off-target sites and result in potentially new off-target interactions requiring fewer base pairs.

The proteins bound to the SOD-1 minigene mRNA more effectively inhibited off-target ASO binding than on on-target ASO binding (Fig. 5B and Table 6). For example, the K_d_s for ASOs 37, 38, 40, and 82 for off-target sites absent proteins (e.g., mRNA spiked into the denatured nuclear extract) ranged from 10 to 37 nM compared to the 44 to 672 nM K_d_s observed for these ASOs bound to the spliced mRNA in extract containing the RNA-binding proteins (Tables 5 and 6). These results suggest that the reductions in affinity of the ASOs at the off-target sites were sufficient to render the ASOs ineffective at competing with proteins for binding to the mRNA. The fact that modest reductions in ASO binding affinity can have a profound effect on the ability of ASOs to compete with both higher order RNA structure and protein for binding to the mRNA demonstrates that the binding affinities observed for the on-target ASO interactions are at or near the threshold required to compete effectively with these factors for binding to the mRNA. Again, incorporating higher affinity nucleotide modifications would enhance the ability of ASOs to compete with proteins for binding to the target RNA, but given that the on-target ASO interactions were effective at competing with proteins for binding to the mRNA, higher affinity nucleotide modifications would likely have a modest effect on on-target hybridization but lead to more off-target interactions.

Human RNase H1 is involved in the ASO-mediated degradation of mRNA targets in human cells [4]. Increasing the levels of human RNase H1 in cells increases the potency of ASOs, whereas decreasing the levels of the enzyme decrease ASO potency, suggesting that human RNase H1 is likely the rate-limiting step with respect to ASO activity in cells [4]. Absent knowledge of the specific kinetic parameters by which human RNase H1 cleaves ASO-RNA heteroduplexes in cells, we determined the on- and off-target cleavage activities for human RNase H1 using enzyme concentrations either in excess (single-turnover kinetics) or below (multiple-turnover kinetics) the substrate concentration (Fig. S2D). Under single-turnover conditions human RNase H1 cleaved the on- and off-target heteroduplexes with similar efficiencies (Table S1). Conversely, under multiple-turnover conditions in which the enzyme concentration was limiting, efficient cleavage of the on-target heteroduplexes and no measurable cleavage of the off-target heteroduplexes were observed (Table S1). These results suggest that if RNase H1 in cells functions under single-turnover kinetics, the off-target ASO binding observed for the SOD-1 minigene mRNA will result in cleavage of the mRNA in cells. If, on the other hand, RNase H1 in cells function under multiple-turnover kinetics the observed off-target heteroduplexes would not be substrates for the enzyme. Certain ASOs bound to their respective off-target sites in cells as degradation of the mutant SOD-1 minigene mRNA containing only the off-target sites were observed in cells overexpressing *E. coli* RNase H1 (Fig. 8 and 9). The fact that no off-target ASO activity was observed for the SOD-1 minigene mRNA in wild-type cells expressing only endogenous human RNase H1 suggests that human RNase H1 activity in cells likely function under multiple-turnover kinetics (Fig. 8 and 9).

The correspondence between ASO cleavage efficiencies of the SOD-1 minigene mRNA that was transcribed and spliced in the nuclear extract and the SOD-1 minigene mRNA in cells was impressive (Fig. 6). The nuclear extract does not contain the cytoplasmic factors involved in mRNA biogenesis. Thus, our data suggest that either the majority of the ASO activity in cells takes place in the nucleus or that the cytoplasmic mRNA binding proteins exhibit binding affinities similar to those observed for the nuclear proteins and that the higher order structure of the mRNA is fairly well conserved throughout its biogenesis. The off-target ASO activity observed for the SOD-1 minigene mRNA in cells overexpressing *E. coli* RNase H1 showed that the ASO interactions targeted the same region within the mRNA that was identified using the naked mRNA (Fig. 2, 8, 9 and Fig. S4A). Again, the correlations between the cell and cell-free systems indicate similar ASO accessibility with respects to higher order structure of the mRNA and RNA binding protein interactions for both the on and off-target sites.

Taken together these results show that the ASO configuration tested here (e.g., ten deoxyribonucleotides flanked with ten 2′-methoxyethylribonucleotides) have binding affinities that both minimize off-target interactions and are sufficient to compete effectively with higher order structure of the RNA and RNA binding proteins. These data further suggest that the threshold for achieving robust off-target ASO activity is high and requires an accessible target site (e.g., single-stranded region absent RNA binding proteins), sufficient base complementarity to achieve binding affinities similar to on-target heteroduplexes (e.g., >13 base-pairs), and sufficient base complementarity within the deoxyribonucleotide region to support human RNase H1 activity. Our identification of the factors that influence the on- and off-target activity of ASOs will make it possible to design ASO configurations that are both more potent and more specific. This study focused on the mRNA; characterization of the interactions of ASOs with the pre-mRNA is also important and will be the focus of future studies.

## Supporting Information

Figure S1 **Identification of proteins in nuclear extract bound to the SOD-1 minigene mRNA.** (A) The biotinylated capture oligonucleotide was annealed to the T7 SOD-1 minigene mRNA in the nuclear extract and the mRNA was captured using magnetic beads. The bound proteins were displaced from the captured mRNA using complementary ASOs shown in panel B and analyzed by western blot. (B) The 20-nucleotide ASOs contained uniform phosphodiester linkages with10 deoxynucleotides (d) flanked by five 2′-methoxyethylribonucleotides (e) at the 5′ and 3′ termini of the ASO. The hybridization sites for the ASOs were designed to cover the entire exon 4 (blue sequence) and exon 5 (black sequence) regions with the ASO target sites overlapping by 10 nucleotides. (C) Identified proteins included H-complex proteins hnRNP H, F, A1, and A1B2; E-complex splicing factors SF2, U1, SF1, and SFRS5; exon-junction complex proteins Magoh, UAF35, UAF56, Y14, and Aly; and RNA binding proteins (RNA-BP) nucleolin (nuc) and DHX36. Proteins were identified using western blot analysis. Red boxes (+) indicate proteins identified following displacement from the mRNA by the respective ASO. Blue boxes (-) indicate protein not identified following displacement from the mRNA by the respective ASO.
(EPS)

Figure S2 **Protocols for analysis of ASO/target mRNA duplex formation.** (A) The labeled T7 transcript was hybridized with ASO and digested with excess *E. coli* RNase H1. The digestion reactions were separated on a denaturing polyacrylamide gel and analyzed using a phosphorimager. Given that the cleavage reactions were performed using excess *E. coli* RNase H1, the level of cleavage activity corresponds to the amount of ASO/mRNA heteroduplex formed. (B) The T7 transcript in denatured nuclear extract was hybridized with ASO and digested with excess *E. coli* RNase H1. The purified mRNA was analyzed by quantitative RT-PCR. The level of cleavage activity corresponds to the amount of ASO/mRNA heteroduplex formed. (C) The mRNA processed in the nuclear extract was hybridized with ASO and digested with excess *E. coli* RNase H1. The purified mRNA was analyzed by quantitative RT-PCR. The level of cleavage activity corresponds to the amount of ASO/mRNA heteroduplex formed. (D) The T7 transcript in denatured nuclear extract was hybridized with high concentration of ASO to ensure all the mRNA was bound with ASO and then digested with either excess *E. coli* or human RNase H1 or limiting concentrations of the human enzyme. On- and off-target cleavage activity was analyzed by qRT-PCR using, respectively, primers amplifying exon 5 and exon 4.
(EPS)

Figure S3 **Binding affinities of ASOs for the SOD-1 minigene mRNA spiked into the denatured nuclear extract.** The percent mRNA cleaved corresponds to the percent mRNA bound to ASO and was plotted as a function of the ASO concentration. The K_d_ was defined as the ASO concentration resulting in 50% mRNA cleaved (i.e., bound to ASO). The mean and errors reported are based on three trials.
(EPS)

Figure S4 **Identification of off-target binding sites in the SOD-1 minigene mRNA.** (A) On-target binding sites for ASOs 37, 38, 40, and 82 (blue lines) targeting exon 5 (black sequence) and predicted off-target sites (red lines) in exon 4 (blue sequence). (B) Predicted off-target heteroduplexes and free energies for hybridization calculated using RNAstructure 5.3. (C) Enzymatic structure mapping of the ASOs (orange and black sequences) hybridized to their respective off-target oligoribonucleotides (red sequences). The red and blue arrows represent the cleavage sites observed for the single-strand specific RNases A and T1, respectively, which cleave the phosphate bond downstream of guanosine and purines, respectively. Blue lines indicate predicted base pairing between the ASOs and oligoribonucleotide targets based on the RNase cleavage patterns.
(EPS)

Figure S5 **Overexpression of** ***E. coli*** **RNase H1 in the SOD1/TO HeLa cells.** *E. coli* RNase H ASKA clone, JW0204 (GFP minus), was obtained NAIST (Nata-ken Japan). The full-length gene was amplified by PCR using PfuTurbo DNA Polymerase (Agilent Technologies) with FP- CACCCATGCTTAAACAGGTAGA and RP- ACCTTCAACTTGGTAGCCTGT. The blunt end PCR product was then TOPO cloned into pcDNA3.1D/V5-His-TOPO according to the manufacturer's protocol (Invitrogen). Plasmid transfection was carried out as described above with stable transformants selected using 800 µg/mL geneticin. A) Geneticin-resistant colonies were expanded, then tested for RNase H expression by western blot performed as described elsewhere [32]. The mouse monoclonal antibody to V5 was obtained from Invitrogen (R960-25) and mouse anti-γ-tubulin from Sigma-Aldrich (T5326). B) RNase H cleavage assays were performed with extracts prepared from SOD1/TO and SOD1/TO-RHA cells. Briefly, ∼10^7^ cells were pelleted, lysed 30 minutes in 1 mL 6 M Gu-HCl and 100 mM NaHPO_4_, pH 8, and 100 µL Ni-NTA agarose (Qiagen) was added to the lysate and incubation continued for 30 minutes. The resin was then washed with 8 M urea, 20 mM imidizol, 100 mM NaHPO_4_, pH 6.3. Resin-bound RNase H was renatured by subsequent washes of 6, 4, 2, 1, and 0.5 M urea in the same buffer. Finally, resin-bound RNase H was suspended in 100 µL 100 mM NaCl, 10 mM Tris-HCl, pH 6.3. An RNA oligoribonucleotide was 5′-end-labeled with 32P using [γ-32P]ATP and T4 polynucleotide kinase, then the heteroduplex substrate prepared as previously described [33]. An aliquot of 100 nM of the substrate was added to the resin-bound RNase H in a total volume of 100 µL cleavage buffer (20 mM Tris-HCl, 50 mM NaCl, 2 mM MgCl_2_, and 0.1 mM TCEP, pH 7.5). Aliquots of 10 µL of the cleavage reaction were removed at time points ranging from 1.5 to 120 min and were quenched by addition of 5 µL of stop solution (8 M urea and 500 mM EDTA). The aliquots were heated at 90°C for 2 min, resolved on a 12% denaturing polyacrylamide gel, and the substrate and product bands were quantitated on an Amersham Biosciences PhosphorImager.
(EPS)

Figure S6 **Effect of human RNase H1 overexpression on antisense activity.** HeLa cells harboring the SOD/TO or the SOD 282_DL minigene were infected with adenoviral human H1 for 48 hours [4]. Cells were seeded in 96-well plates then treated with ASO at concentrations between 0.5 and 150 nM. Following transfection and TET induction of the minigene, target RNA reduction was assayed by qRT/PCR. Data are presented as percent mock-transfected control for control (solid line) and adenoviral-infected cells (dashed line). A) Western blot of adenoviral infected cells using RNase H1 polyclonal antibody. B) SOD/TO cell line representing target specific cleavage activity. C) SOD 282_DL cells line representing off-target cleavage.
(EPS)

Table S1 **Human RNase H1 cleavage activity for on- and off-target ASO binding to the SOD-1 minigene mRNA spiked into the denatured nuclear extract.** Human RNase H1 cleavage activity is reported as percent mRNA reduction. Cleavage of mRNA hybridized with ASOs 37, 38, 40, and 82 to on-target and off-target sites using excess *E. coli* RNase H1, excess human RNase H1 or limiting human RNase H1. The mean and errors reported are based on three trials.
(EPS)

## References

[1] Crooke ST (2003) In Burger's Medicinal Chemistry (Abraham, D. J., ed) 6^th^ ED. Vol. 5 , pp115–166, John Wiley and Sons, Inc., New York.

[2] Crooke ST (1999) Molecular mechanisms of antisense drugs. Biochem Biophys Acta, 1489 (1): , 31–43. 10.1016/s0167-4781(99)00148-7 10806995

[3] Crooke ST (2001) In Antisense Technology: Principles, Strategies, and Applications (Crooke, S. T., ed) pp. 1–28, Marcel Dekker, Inc., New York.

[4] Wu H, Lima WF, Zhang H, Fan A, Sun H, et al. (2004) Determination of the role of the human RNase H1 in the pharmacology of DNA-like antisense drugs. J Biol Chem, 279 (17): , 17181–17189. 10.1074/jbc.M311683200 14960586

[5] Itaya M, Kondo K (1991) Molecular cloning of a ribonuclease H (RNase HI) gene from an extreme thermophile Thermus thermophilus HB8: a thermostable RNase H can functionally replace the Escherichia coli enzyme in vivo. Nucleic Acids Res, 19 (16): , 4443–4449. 10.1093/nar/19.16.4443 PMC328632 1653414

[6] Itaya M, McKelvin D, Chatterjee SK, Crouch RJ (1991) Selective cloning of genes encoding RNase H from *Salmonella typhimurium*, *Saccharomyces cerevisiae* and *Escherichia coli rnh* mutant. Mol Gen Genet, 227 (3): , 438–445. 10.1007/BF00273935 1650910

[7] Kanaya S, Itaya M (1992) Expression, purification, and characterization of a recombinant ribonuclease H from *Thermus thermophilus* HB8. J Biol Chem, 267 (14): , 10184–10192. 1315754

[8] Busen W (1980) Purification, subunit structure, and serological analysis of calf thymus ribonuclease H1. J Biol Chem, 255 (19): , 9434–9443. 6251088

[9] Rong YW, Carl PL (1990) On the molecular weight and subunit composition of calf thymus ribonuclease H1. Biochemistry, 29 (2): , 383–389. 10.1021/bi00454a012 2154245

[10] Eder PS, Walder JA (1993) Substrate specificity of human RNase H1 and its role in excision repair of ribose residues misincorporated in DNA. Biochimie, 75 (1): , 6472–6479. 10.1016/0300-9084(93)90033-o 8389211

[11] Vickers TA, Koo S, Bennett CF, Crooke ST, Dean NM, et al. (2003) Efficient reduction of target RNAs by small interfering RNA and RNase H-dependent antisense agents. A comparative analysis. J Biol Chem, 278 (9): , 7108–7118. 10.1074/jbc.M210326200 12500975

[12] Bennett CF, Swayze EE (2010) RNA Targeting Therapeutics: Molecular Mechanisms of Antisense Oligonucleotides as a Therapeutic Platform. Annual Review of Pharmacology and Toxicology, 50 (1): , 259–293. 10.1146/annurev.pharmtox.010909.105654 20055705

[13] Swayze EE, Bhat B (2007) In Antisense Drug Technology Principles, Strategies, and Applications (Crroke S. T., ed) 2^nd^ ED. pp 143–182, CBC press, New York.

[14] Monia BP, Johnston JF, Ecker DJ, Zounes MA, Lima WF, et al. (1992) Selective inhibition of mutant Ha-ras mRNA expression by antisense oligonucleotides. J Biol Chem, 267 (28): , 19954–19962. 1400312

[15] Monia BP, Lesnik EA, Gonzalez C, Lima WF, McGee D, et al (1993) Evaluation of 2'-modified oligonucleotides containing 2'-deoxy gaps as antisense inhibitors of gene expression. J Biol Chem, 268 (19): , 14514–14522. 8390996

[16] Baker BF, Lot SS, Condon TP, Cheng-Flournoy S, Lesnik EA, et al. (1997) 2'-O-(2-Methoxy)ethyl-modified anti-intercellular adhesion molecule 1 (ICAM-1) oligonucleotides selectively increase the ICAM-1 mRNA level and inhibit formation of the ICAM-1 translation initiation complex in human umbilical vein endothelial cells. J Biol Chem, 272: , 11994–12000. 10.1074/jbc.272.18.11994 9115264

[17] Wagner RW, Matteucci MD, Lewis JG, Gutierrez AJ, Moulds C, et al. (1993) Antisense gene inhibition by oligonucleotides containing C-5 propyne pyrimidines. Science, 260 (5113): , 1510–1513. 10.1126/science.7684856 7684856

[18] Monia BP, Sasmor H, Johnston JF, Freier SM, Lesnik EA, et al. (1996) Sequence-specific antitumor activity of a phosphorothioate oligodeoxyribonucleotide targeted to human C-raf kinase supports an antisense mechanism of action in vivo. Proc Natl Acad Sci U S A, 93 (26): , 15481–15484. 10.1073/pnas.93.26.15481 PMC26430 8986837

[19] Freier SM, Altmann KH (1997) The ups and downs of nucleic acid duplex Structure-stability studies on chemically-modified DNA:RNA duplexes. Nucleic Acids Res, 25 (22): , 4429–4443. 10.1093/nar/25.22.4429 PMC147101 9358149

[20] Lima WF, Monia BP, Ecker DJ, Freier SM (1992) Implication of RNA structure on antisense oligonucleotide hybridization kinetics. Biochemistry, 31 (48): , 12055–12061. 10.1021/bi00163a013 1280997

[21] Mathews DH, Burkard SM, Freier SM, Wyatt JR, Turner DH (1999) Predicting oligonucleotide affinity to nucleic acid targets. RNA, 5 (11): , 1458–1469. 10.1017/s1355838299991148 PMC1369867 10580474

[22] Freier SM, Watt AT (2007) In Antisense Drug Technology Principles, Strategies, and Applications (Crroke S. T., ed) 2^nd^ ED. pp 118–138, CBC press, New York.

[23] Baughan TD, Dickson A, Osman EY, Lorson CL (2009) Delivery of bifunctional RNAs that target an intronic repressor and increase SMN levels in an animal model of spinal muscular atrophy. Hum Mol Genet, 18 (9): , 1600–1611. 10.1093/hmg/ddp076 PMC2667287 19228773

[24] Cartegni L, Krainer AR (2003) Correction of disease-associated exon skipping by synthetic exon-specific activators. Nat Struct Mol Biol, 10 (2): , 120–125. 10.1038/nsb887 12524529

[25] Skordis LA, Dunckley MG, Yue B, Eperon IC, Muntoni F (2003) Bifunctional antisense oligonucleotides provide a trans-acting splicing enhancer that stimulates *SMN2* gene expression in patient fibroblasts. Proc Natl Acad Sci USA, 100 (7): , 4114–4119. 10.1073/pnas.0633863100 PMC153057 12642665

[26] Villemaire J, Dion I, Elela SA, Chabot B (2003) Reprogramming alternative pre-messenger RNA splicing through the use of protein-binding antisense oligonucleotides. J Biol Chem, 278 (50): , 50031–50039. 10.1074/jbc.M308897200 14522969

[27] Goraczniak R, Behlke MA, Gunderson SI (2009) Gene silencing by synthetic U1 adaptors. Nat Biotechnol, 27 (3): , 257–263. 10.1038/nbt.1525 19219028

[28] Rigo F, Hua Y, Chun SJ, Prakash TP, Krainer AR, et al. (2012) Synthetic oligonucleotides recruit ILF2/3 to RNA transcripts to modulate splicing. Nat Chem Biol, 8 (6): , 555–561. 10.1038/nchembio.939 PMC5021312 22504300

[29] Lee J, Lee H-J, Shin M-K, Ryu W-S (2004) Versatile PCR-mediated insertion or deletion mutagenesis. Biotechniques, 36 (3): , 398–400. 10.2144/04363BM04 15038153

[30] Mckay R, Miraglia L, Cummins L, Owens S, Sasmor HM, et al. (1999) Characterization of a potent and specific class of antisense oligonucleotide inhibitor of human protein kinase C-α expression. J Biol Chem, 274 (3): , 1715–1722. 10.1074/jbc.274.3.1715 9880552

[31] Sambrook J, Fritsch EF, Maniatis T (1989) In Molecular Cloning. A Laboratory Manual, 2^nd^ ed., Cold Spring Harbor Laboratory Press, Cold Spring Harbor, NY.

[32] Vickers TA, Lima WF, Nichols JG, Crooke ST (2007) Reduced levels of Ago2 expression result in increased siRNA competition in mammalian cells. Nucleic Acids Res, 35 (19): , 6598–6610. 10.1093/nar/gkm663 PMC2095815 17905815

[33] Lima WF, Wu H, Nichols JG, Manalili SM, Drader JJ, et al. (2003) Human RNase H1 activity is regulated by a unique redox switch formed between adjacent cysteines. J Biol Chem, 278 (17): , 14906–14912. 10.1074/jbc.M211279200 12473655

[34] Vickers TA, Zhang H, Graham MJ, Lemonidis KM, Zhao C, et al. (2006) Modification of MyD88 mRNA Splicing and Inhibition of IL-1beta Signaling in Cell Culture and in Mice with a 2'-O-Methoxyethyl-Modified Oligonucleotide. J Immunology, 176 (6): , 3652–3661. 10.4049/jimmunol.176.6.3652 16517734

[35] Winer J, Kwang C, Jung S, Shackel I, Williams PM (1999) Development and Validation of Real-Time Quantitative Reverse Transcriptase±Polymerase Chain Reaction for Monitoring Gene Expression in Cardiac Myocytes in Vitro. Anal Biochem, 270 (1): , 41–49. 10.1006/abio.1999.4085 10328763

[36] Hashimoto JG, Beadles-Bohling AS, Wiren KM (2004) Comparison of RiboGreen and 18S rRNA quantitation for normalizing real-time RT-PCR expression analysis. Biotechniques, 36 (1): , 58–60. 10.2144/04361BM06 14740484

[37] Kataoka N, Yong J, Kim VN, Velazquez F, Perkinson RA, et al. (2000) Pre-mRNA splicing imprints mRNA in the nucleus with a novel RNA-binding protein that persists in the cytoplasm. Mol Cell, 6 (3): , 673–682. 10.1016/s1097-2765(00)00065-4 11030346

[38] Le Hir H, Gatfield D, Izaurralde E, Moore MJ (2001) The exon-exon junction complex provides a binding platform for factors involved in mRNA export and NMD. EMBO J, 20 (17): , 4987–4997. 10.1093/emboj/20.17.4987 PMC125616 11532962

[39] Le Hir H, Izaurralde E, Maquat LE, Moore MJ (2000) The spliceosome deposits multiple proteins 20–24 nucleotides upstream of mRNA exon-exon junctions. EMBO J, 19 (24): , 6860–6869. 10.1093/emboj/19.24.6860 PMC305905 11118221

[40] Jamison SF, Crow A, Garcia-Blanco MA (1992) The spliceosome assembly pathway in mammalian extracts. Mol Cell Biol, 12 (10): , 4279–4287. 10.1128/mcb.12.10.4279-4287.1992 PMC360351 1383687

[41] Seraphin B, Rosbash M (1989) Identification of functional U1 snRNA pre-messenger RNA complexes committed to spliceosome assembly and splicing. Cell, 59 (2): , 349–358. 10.1016/0092-8674(89)90296-1 2529976

[42] Ghisolfi-Nieto L, Joseph G, Puvion-Dutilleul F, Amalric F, Bouvet P (1996) Nucleolin is a Sequence-specific RNA-binding Protein: Characterization of Targets on Pre-ribosomal RNA. J Mol Biol, 260 (1): , 134–153. 10.1006/jmbi.1996.0380 8676391

[43] Abdelhaleem M, Maltais L, Wain H (2003) The human DDX and DHX gene families of putative RNA helicases. Genomics, 81 (6): , 618–622. 10.1016/s0888-7543(03)00049-1 12782131

[44] Chou MY, Rooke N, Turck CW, Black DL (1999) hnRNP H is a component of a splicing enhancer complex that activates a c-src alternative exon in neuronal cells. Mol Cell Biol, 19 (1): , 69–77. 10.1128/mcb.19.1.69 PMC83866 9858532

[45] Yang X, Bani MR, Lu SJ, Rowan S, Ben-David Y et al. (1994) The A1 and A1B proteins of heterogeneous nuclear ribonucleoparticles modulate 5' splice site selection in vivo. Proc Natl Acad Sci U S A, 91: , 6924–6928. 10.1073/pnas.91.15.6924 PMC44310 8041722

[46] Caputi M, Zahler AM (2001) Determination of the RNA Binding Specificity of the Heterogeneous Nuclear Ribonucleoprotein (hnRNP) H/H′/F/2H9 Family. J Biol Chem, 276 (47): , 43850–43859. 10.1074/jbc.M102861200 11571276

[47] Reichert VL, Le Hir H, Jurica MS, Moore MJ (2002) 50 exon interactions within the spliceosome establish a framework for exon junction complex structure and assembly. Genes Dev, 16: , 2778–2791. 10.1101/gad.1030602 PMC187475 12414731

[48] Shibuya T, Tange TO, Sonenberg N, Moore MJ (2004) eIF4AIII binds spliced mRNA in the exon junction complex and is essential for nonsense mediated decay. Nat Struct Mol Biol, 11 (4): , 346–351. 10.1038/nsmb750 15034551

[49] Yao F, Svensjo T, Winkler T, Lu M, Eriksson C, Eriksson E (1998) Tetracycline repressor, tetR, rather than the tetR-mammalian cell transcription factor fusion derivatives, regulates inducible gene expression in mammalian cells. Hum Gene Ther, 9: , 1939–1950. 10.1089/hum.1998.9.13-1939 9741432

[50] Seth PP, Jazayeri A, Yu J, Allerson CR, Bhat B, et al. (2012) Structure Activity Relationships of alpha-L-LNA Modified Phosphorothioate Gapmer Antisense Oligonucleotides in Animals. Mol Ther Nucleic Acids, 1: , e47. 10.1038/mtna.2012.34 PMC3499693 23344239

[51] Lima WF, Brown-Driver V, Fox M, Hanecak R, Bruice TW (1997) Combinatorial screening and rational for hybridization to folded Hepatitis C virus RNA of oligonucleotides with biological antisense activity. J Biol Chem, 272 (1): , 626–638. 8995306

[52] Arhin GK, Boots M, Bagga PS, Milcarek C, Wilusz J (2002) Downstream sequence elements with different affinities for the hnRNP H/H′ protein influence the processing efficiency of mammalian polyadenylation signals. Nucleic Acids Res, 30 (8): , 1842–1850. 10.1093/nar/30.8.1842 PMC113221 11937639

